# Deep Learning Recurrent Neural Network for Concussion Classification in Adolescents Using Raw Electroencephalography Signals: Toward a Minimal Number of Sensors

**DOI:** 10.3389/fnhum.2021.734501

**Published:** 2021-11-24

**Authors:** Karun Thanjavur, Dionissios T. Hristopulos, Arif Babul, Kwang Moo Yi, Naznin Virji-Babul

**Affiliations:** ^1^Department of Physics and Astronomy, University of Victoria, Victoria, BC, Canada; ^2^School of Electrical and Computer Engineering, Technical University of Crete, Chania, Greece; ^3^Department of Computer Science, University of British Columbia, Vancouver, BC, Canada; ^4^Djavad Mowafaghian Centre for Brain Health, University of British Columbia, Vancouver, BC, Canada; ^5^Department of Physical Therapy, Faculty of Medicine, University of British Columbia, Vancouver, BC, Canada

**Keywords:** concussion, mild traumatic brain injury, resting state EEG, adolescents, machine learning, deep learning, LSTM, concussion classification

## Abstract

Artificial neural networks (ANNs) are showing increasing promise as decision support tools in medicine and particularly in neuroscience and neuroimaging. Recently, there has been increasing work on using neural networks to classify individuals with concussion using electroencephalography (EEG) data. However, to date the need for research grade equipment has limited the applications to clinical environments. We recently developed a deep learning long short-term memory (LSTM) based recurrent neural network to classify concussion using raw, resting state data using 64 EEG channels and achieved high accuracy in classifying concussion. Here, we report on our efforts to develop a clinically practical system using a minimal subset of EEG sensors. EEG data from 23 athletes who had suffered a sport-related concussion and 35 non-concussed, control athletes were used for this study. We tested and ranked each of the original 64 channels based on its contribution toward the concussion classification performed by the original LSTM network. The top scoring channels were used to train and test a network with the same architecture as the previously trained network. We found that with only six of the top scoring channels the classifier identified concussions with an accuracy of 94%. These results show that it is possible to classify concussion using raw, resting state data from a small number of EEG sensors, constituting a first step toward developing portable, easy to use EEG systems that can be used in a clinical setting.

## Introduction

One of the most significant challenges in the diagnosis of concussion (mild traumatic brain injury) is the lack of objective, clinically accepted, brain-based approaches for determining whether an individual has experienced a concussion. Currently, the diagnosis of concussion relies on subjective reporting of the signs and symptoms from individual patients. However, it is becoming increasingly clear that symptoms do not directly correlate to the underlying changes in brain structure and function and that changes in the brain persist well beyond symptomatic recovery (Manning et al., 2017; Churchill et al., 2019). Importantly, individuals who self-report their first concussion during childhood have a higher risk of sustaining additional concussions throughout their lifetime (Schmidt et al., 2018), and higher risk of medical impairments and altered social functioning in adulthood (Sariaslan et al., 2016). Given that the lack of, or delay in, concussion diagnosis is associated with much slower recovery (Asken et al., 2018), there is an urgent need for an objective, low cost, brain-based measure of concussive brain injury.

There is extensive literature documenting changes in brain functional activity that occur following concussion in both resting state and task-based electroencephalography (EEG) (see Conley et al., 2019 for review). Two of the main limitations of EEG analysis are the time required and the need for specialized expert knowledge to identify features in the EEG signal. Recently, machine learning approaches such as support vector machine (SVM), random forest (e.g., Cao and Slobounov, 2010; Munia et al., 2017; Vergara et al., 2017; Jacquin et al., 2018; Wickramaratne et al., 2020) as well as deep learning, convolution neural network (Boshra et al., 2019) have been used to classify concussion using both resting state and task-based EEG signals with varying degrees of success. The main issue with these previous methods is that they all require significant preprocessing of the time series. Typically, the acquired EEG data are filtered and cleaned to remove these artifacts (c.f. Munia et al., 2017; Hristopulos et al., 2019), with cleaning strategies in use ranging from fully automated algorithms to mixed schemes where some artifacts are removed via automated algorithms and others are manually identified and removed.

**FIGURE 1:**
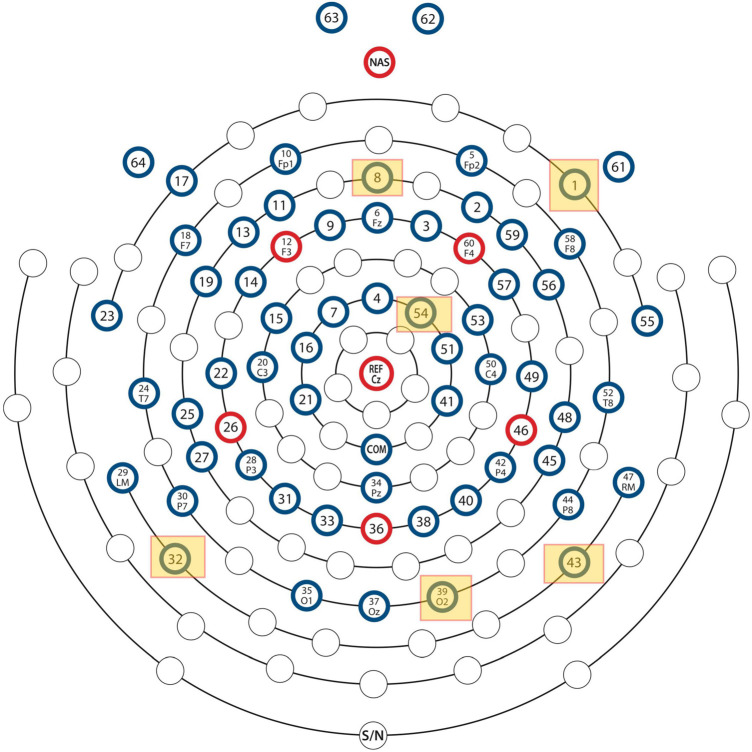
Locations of the EEG electrodes on the scalp. The locations of the six most important channels with respect to classification performance of the neural network are marked by yellow squares.

We have recently developed and reported on *ConcNet 2*, a deep learning long short-term memory (LSTM)-based recurrent neural network that is able to distinguish between non-concussed and acute post-concussed adolescent athletes using only short (i.e., 90 s long) samples of raw, resting state data from 64 sensors, distributed over the scalp as shown in Figure 1, as input. We refer readers to Thanjavur et al. (2021) for details, including the reasons for deliberately choosing to work with raw data. In short, the latter strategy was adopted to circumvent concerns about pre-processing (including possible signal distortions that can arise as a result of re-referencing as well as filtering and cleaning the data) and instead rely on the deep learning network to discover the relevant aspects of the data while ignoring redundant and/or immaterial information. By using raw data, we also avoid having to grapple with the complex problem of feature selection (Munson and Caruana, 2009; Krawczuk and Łukaszuk, 2016; Neumann et al., 2016), which in itself can be a source of bias (Sahiner et al., 2000; Smialowski et al., 2009; Krawczuk and Łukaszuk, 2016). In addition, we avoid confounding effects on potential feature values due to the EEG signals’ strongly non-stationary character in both the temporal and the spectral domains (Kaplan et al., 2005; Klonowski, 2009; Cao and Slobounov, 2011). During rigorous testing, the LSTM-based classifier (henceforth *ConcNet 2)* consistently identified concussions with an accuracy of >90% and achieved an ensemble median Area Under the Receiver Operating Characteristic Curve (ROC/AUC) equal to 0.971 (Thanjavur et al., 2021). This is the first instance of a high-performing classifier that relies only on easy-to-acquire resting state, raw EEG data.

The *ConcNet 2* classifier was a key first step toward the development of an easy-to-use, objective, brain-based, automatic classification of concussion at an individual level. An important next step is to reduce the number of required EEG channels while maintaining or even enhancing the performance of the classifier to develop an EEG based system that is easier to use in clinical settings. This is motivated by the fact that certain EEG channels provide redundant information. Therefore, research efforts are underway to determine the minimum number of channels required in both resting state and task-based conditions. Recently, Moctezuma and Molinas (2020) reported on a proof-of-concept study showing that using a dataset of 64 channels from resting state with eyes closed, they were able to obtain a true acceptance rate of up to 0.997 using discrete wavelet transform (DWT) based features with only three channels.

The goal of the present study is to describe the process of selection and the performance of a minimal subset of the original 64 channel dataset (using the same participants as in Thanjavur et al., 2021) that leads to the same level of accuracy in classifying acute concussion as the entire 64 channel dataset.

## Materials and Methods

### Participants

Fifty-eight male adolescent athletes volunteered for this study. Of these, 23 individuals were athletes who had suffered a sport-related concussion (mean = 13.4, *SD* = 2.5) and 35 were non-concussed, control athletes (mean = 14.7, *SD* = 2.1). All participants reported normal or corrected to normal vision. Individuals with focal neurologic deficits, pathology and/or those on prescription medications for neurological or psychiatric conditions were excluded from this study. All participants who had braces or permanent retainers were also excluded. The study was approved by the University of British Columbia Clinical Research Ethics Board (Approval number: H17-02973). All participants provided assent and the adolescents’ parents gave written informed consent for their children’s participation under the approval of the Human Ethics Review Board of the University of British Columbia in accordance with the Helsinki declaration.

### Clinical Assessment

All concussed athletes had a clinical assessment by the team physician or a physician with expertise in concussion within a week of injury, and they were found to meet the concussion diagnostic criteria consistent with the Berlin consensus statement (McCrory et al., 2017). The latter includes: (1) Documentation of the date and time of the injury by the team coach. Here, injury refers to either a direct blow to the head, face, or neck, or a direct blow to the body that leads to an impulsive force transmitted to the head, resulting in changes in one or more of the following clinical domains: (a) physiological (e.g., neck pain, balance problems, headache, fatigue), (b) cognitive (e.g., difficulty with attention, feeling in a “fog”), (c) emotional (e.g., irritability, sadness, depression) and (d) behavioral (i.e., sleep/wake disturbances). (2) An assessment at the time of diagnosis of the injured athlete using either the Child Sports Concussion Assessment Tool 3 (Child SCAT3), if the injured athlete was younger than 13 years of age (“Child SCAT3.” 2013), or using the Sports Concussion Assessment Tool-3 (SCAT-3), if 13 years or older (Chin et al., 2016). Both SCAT-3 and Child SCAT-3 are standardized concussion and concussion symptom assessment tools, which combine self-reporting of the number of symptoms experienced and their severity with cognitive, behavioral, physiological and emotional assessments based on a combination of clinically accepted objective diagnostic tools and self-reporting of symptoms. Finally, the Berlin consensus statement requires a clinical examination as well as a review of all the available information, by an experienced physician.

### Data Acquisition

Five minutes of resting state EEG data were collected from each participant under eyes closed condition using a 64-channel HydroCel Geodesic Sensor Net (EGI, Eugene, OR) connected to a Net Amps 300 high-impedance amplifier with a 0.1 Hz high-pass analog (i.e., hardware) filter. The EGI system uses the vertex (Cz) as physical reference and the signals were recorded at a sampling rate of 250 Hz. Data acquisition was started only after all the scalp electrode impedances were confirmed to be below 50k, in keeping with the recommendations for the EGI Net Amps 300 high-impedance amplifier (Ferree et al., 2001).

EEG data were acquired from the concussed athletes within a month of injury. We chose the time window of 1 month based on the 2017 Berlin Consensus Statement’s expected time for clinical recovery after injury in children and adolescents (McCrory et al., 2017). All of the injured participants were re-assessed using the SCAT3/ChildSCAT3 at the time of data collection, and we acquired data only from those who were symptomatic. The data collection for both the control and concussed groups occurred over a span of 2 years. The recruitment for the study was ongoing during this time frame and participants were invited to participate anytime during this period as long as they met the inclusion/exclusion criteria. EEG data for all participants were collected in the same room/lab at the University of British Columbia using the same equipment and set-up.

### Electroencephalography Data Processing

To eliminate any transients at the start and the end of a data collection session, 1,000 data points were removed from the beginning and the end of each time series. This corresponds to removing data with a total duration of 8 s. The data was neither re-referenced to a different montage nor further filtered or processed to remove artifacts due to line noise, eye blinks and motion, and electromyogram (EMG) contamination. The resting state EEG data were acquired in binary simple (.RAW) format and converted to Matlab (.mat) format using EEGLab (Delorme and Makeig, 2004) for further processing and analysis. Eight 90 s long sets of synchronous segments were extracted from each of the 64-channel time series comprising each individual’s raw resting state EEG dataset.

### Analysis Pipeline

Having successfully developed a high performing concussion classifier using time series data from 64 EEG channels, our present concern is to try to establish whether we can do the same, i.e., develop a concussion classifier, which uses only a small subset of channels, with comparable classification performance as we have achieved with full 64 channels. In general, this is a combinatorial problem, and such problems can always be solved by exploring all possible channel combinations. However, given that we are starting with 64 channels, the associated computational cost is prohibitively expensive. As a workaround, we have opted to use, for exploratory purposes, an algorithm that examines the impact of each channel independently of the others. Specifically, we started with our 64-channel classifier (*ConcNet 2*). We explored the classification efficacy of using one channel only each time. We built up an accuracy score for each of the 64 channels taken one-by-one. We then rank ordered the channels and accepted all channels with accuracy above a selected threshold. This process identified six top-scoring channels.

There is an important caveat to the above approach to take note of. While it is simple, straightforward and intuitive, it does not guarantee that the optimal solution is achieved since it does not account for potential interactions between channels. However, for our purposes it is not necessary to obtain an “optimal solution.” It suffices to determine a reduced subset of channels that can give comparable classification performance as *ConcNet 2*.

The analysis was conducted in two stages as follows: (i) In stage 1, each of the 64 channels was tested and ranked based on its contribution toward the concussion classification performed by *ConcNet 2*. (ii) In stage 2, the top scoring channels were used to train and test a new network, henceforth *ConcNet 3*, with the same RNN architecture as *ConcNet 2*. However, *ConcNet 3*’s input layer was modified to accept 6-channel data (instead of 64 channels). Consequently, three operational *hyperparameters* had to be re-tuned to cope with the 6-channel data. The performance of this network was then compared with the performance of *ConcNet 2* using Monte Carlo cross validation (MCCV) tests.

**FIGURE 2:**
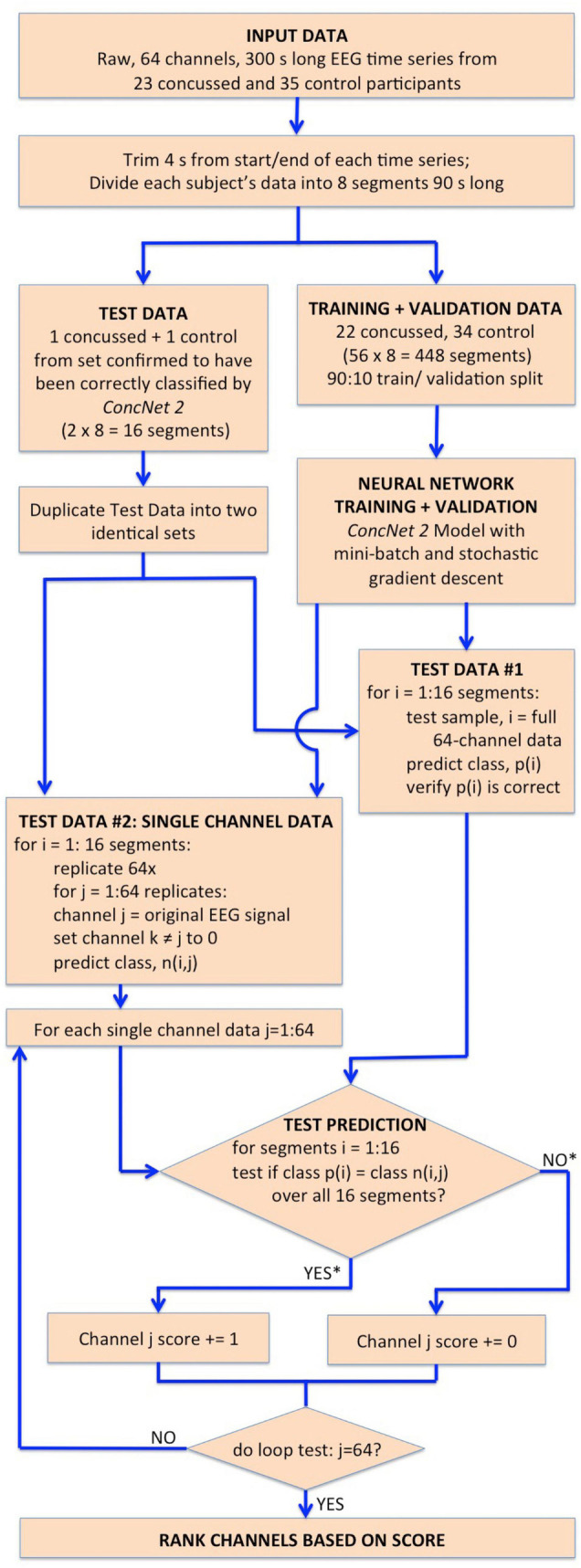
Flowchart illustrating the steps followed in Stage I for the identification of the channels which have the highest impact on the classification performance of *ConcNet 2*. The flowchart begins with the input dataset, followed by the trimming and segmentation of each participant’s data, and then splitting into training, validation and test datasets. *ConcNet 2* was trained and validated using the standard procedure of mini-batch SGD. For Stage I, two test sets are created in parallel as shown, one to benchmark *ConcNet 2*’s performance against earlier results described in Paper I, and the other for ranking the channels by their impact on classification, as explained in the text. As indicated by the asterisks on the outputs of the conditional box for the test prediction, each channel was awarded a score of 1 if and only if the median prediction score ≥ 0.9, and the 2.5th percentile ≥ 0.85, which demanded a high degree of confidence in making a correct classification for all 16 test segments in this test. Finally, this test procedure, and the additional complementary test with three concussed-control pairs described in the text, were each repeated nine times, and the channels ranked based on their total score ranging from 0 to 18, as explained in “Results” section. The six top scoring channels from this overall ranking were taken to be those with the highest impact on the classification performance, and therefore were then used for Stage II of our work.

**FIGURE 3:**
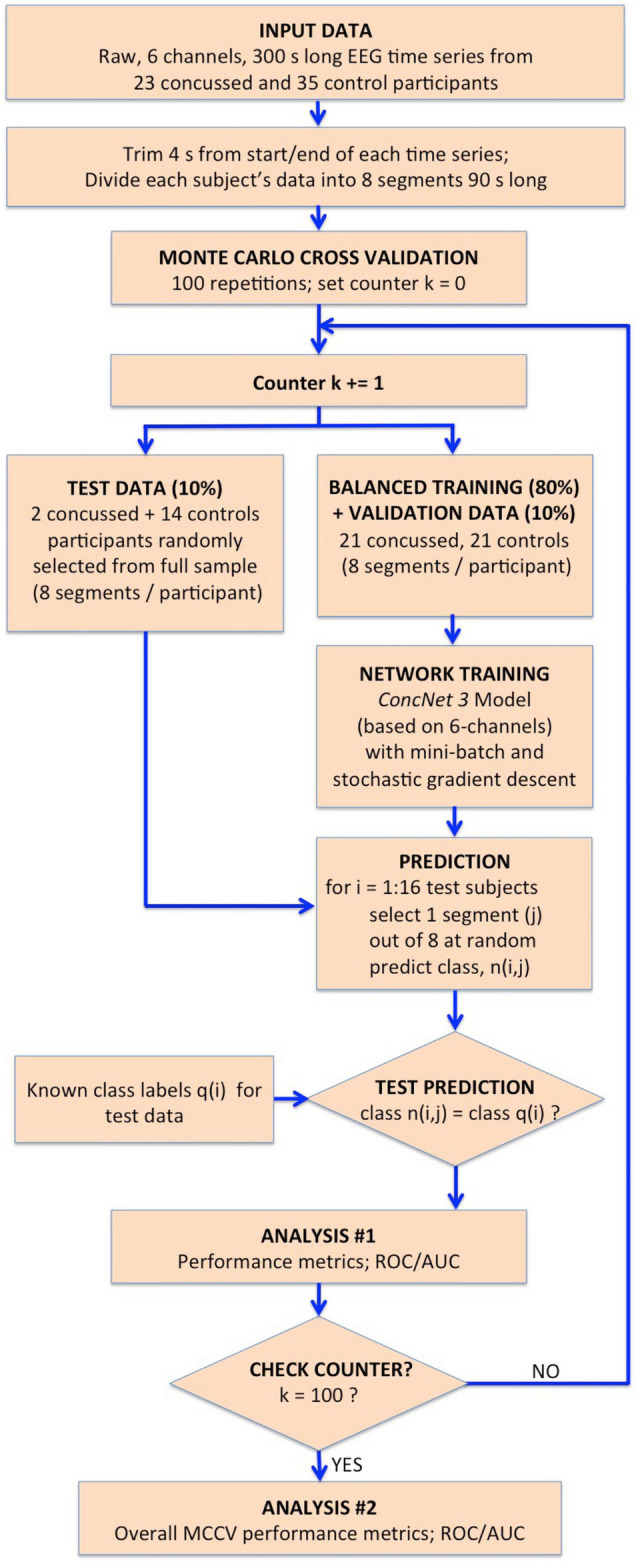
Flowchart illustrating the procedure followed for the MCCV tests in Stage II to compare the performance of *ConcNet 3* using only 6-channel data against that of *ConcNet 2*, which used 64-channel data. As shown, a new dataset was created which contained data for only the six top scoring channels identified in Stage I for all the participants (23 concussed and 35 controls). These data were trimmed and segmented into eight segments for each participant. The dataset was then split in a 80:10:10 ratio into training, validation and test datasets, taking care to ensure the training and validation datasets were balanced between the two classes. *ConcNet 3* was trained and validated using standard mini-batch SGD, after re-tuning three of its hyperparameters, the dropout fraction, learning rate and total number of training epochs to reduce overfitting on the 6-channel data. The predictions made by the trained *ConcNet 3* for the 16 test segments were compared with the known class labels of each segment to obtain the accuracy and other performance metrics for each MCCV cycle. By repeating the cycle 100 times, the median and the quartiles of the accuracy and various other performance metrics reported in Table 2 were obtained, and compared with those of *ConcNet 2* obtained from the MCCV tests reported in Paper I, and reproduced in Table 3 for ease of reference.

The network design, training, validation and testing procedures closely follow those used for *ConcNet 2.* A detailed description of the steps used for the design, training and evaluation of *ConcNet 2* is found in our previous report (Thanjavur et al., 2021), which will henceforth be referred to as Paper I. We have illustrated the procedural steps used in Stage 1 in the flowchart in Figure 2, and those used for the MCCV tests in the flowchart in Figure 3, with full descriptions of these steps in the following subsections.

**TABLE 1:** Demographic information for the participants in the control and concussed groups.

**Demographic information**	**Controls**	**Concussed**
Age (Years, SD)	14.7 (2.1)	13.4 (2.6)
Gender	100% male	100% male
Time since concussion		Between 1 week and 1 month
SCAT (# of symptoms, SD)		8.5 (6.0)
SCAT (symptom severity, SD)		21.0 (21.1)
CHILD scat (# of symptoms, SD)		13.0 (5.4)
CHILD scat (symptom severity, SD)		23.2 (13.6)

**TABLE 2:** Performance metrics computed from the results of the MCCV tests in Stage 2 for the 6-channel data.

**Metric**	**Q_2_**	**Q_1_**	**Q_3_**	**Metric**	**Q_2_**	**Q_1_**	**Q_3_**
Accuracy	0.944	0.889	0.972	Area under curve (AUC)	0.971	0.964	0.978
Recall (TPR)	1.0	1.0	1.0	Informedness	0.933	0.867	0.967
Precision (PPV)	0.75	0.6	1.0	Markedness	0.938	0.882	0.969
Specificity (TNR)	0.933	0.933	1.0	Miss rate (FNR)	0.0	0.0	0.0
False discovery rate (FDR)	0.062	0.0	0.118	False positive rate (FPR)	0.067	0.0	0.067

**TABLE 3:** Comparative performance metrics for *ConcNet 2* computed from the MCCV tests.

**Metric**	**Q_2_**	**Q_1_**	**Q_3_**	**Metric**	**Q_2_**	**Q_1_**	**Q_3_**
Accuracy	0.889	0.833	0.944	Area under curve (AUC)	0.961	0.952	0.969
Recall (TPR)	1.0	0.667	1.0	Informedness	0.867	0.6	0.933
Precision (PPV)	0.667	0.5	0.75	Markedness	0.882	0.646	0.938
Specificity (TNR)	0.933	0.867	0.933	Miss rate (FNR)	0.0	0.0	0.333
False discovery rate (FDR)	0.091	0.082	0.187	False positive rate (FPR)	0.067	0.067	0.133

Stage 1: Identification of Channels with Highest Impact on Classification.

For the concussion classification, our existing neural network, *ConcNet 2* is trained on raw, 64-channel EEG data. Our working hypothesis is that the information content in all the channels is not unique, that some EEG channels provide redundant information. Our aim is to assess and rank the channels by their impact on the classification performance of the network. The procedure we follow in order to accomplish this is detailed in a flowchart shown in Figure 2 and described below.

The acquired data consisted of 300 s long, raw 64-channel EEG time series data from 23 concussed and 35 control participants. Four seconds of data were removed from the start and end of each participant’s 64 EEG time series to remove transient noise signals as the participant settled down and completed the EEG recording. The remaining 292 s-long trimmed time series of each participant was then divided into 8 segments, each 90 s long. Paper I provides the rationale behind this data augmentation, as well as the details of the method used to segment the data. Briefly, of the 8 segments, 3 segments were sequential, beginning at 1, 91, and 181 s and each of length 90 s. The remaining five had random start times but each had a length of 90 s. Therefore, each participant’s data set provided 8 segments of data, which could be used for training, validation and testing the network. We wish to emphasize that *all* 8 segments from each participant went into either the training or the validation or the testing dataset. We thus ensured that the data used to validate or test the network had never been seen during training, and vice versa.

**FIGURE 4:**
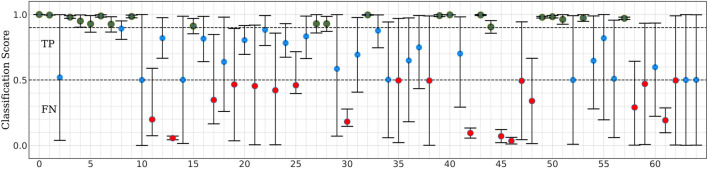
Classification scores of *ConcNet 2* for a pair of one control and one concussed participant based on a total of sixteen data segments.

Next, we split our data into test and training/validation data sets as follows: All eight data segments from one concussed participant, and eight data segments from one control participant were set aside to generate the test set, for a total of 16 segments. This control-concussed pair was randomly selected from the subset of participants, who had been *consistently correctly classified* during the Monte Carlo cross validation (MCCV) testing of *ConcNet 2* reported in our Paper I (c.f. Figure 4 and accompanying text for details). The remainder of the data were split in a 90:10 ratio between the training and validation datasets as described in the flowchart. All eight data segments from 22 concussed and 34 control participants were used for creating the data inputs to the network during training and validation for a total of 448 segments. *ConcNet 2* was then trained using these training and validation datasets.

During this Stage 1 training and validation phase, the hyperparameters of *ConcNet 2* were left unaltered from the values reported in Paper I. For training, we used the standard, mini-batch, stochastic gradient descent (SGD) method. For a detailed description of the procedure, we refer interested readers to Paper I. Here we provide a brief overview: The available 400 segments of training data were split into 20 mini-batches each of 20 segments. For each segment in a mini-batch, the network made a prediction of the class (either concussed or control) based on the state of its internal parameters, namely the weights and biases of the nodes in the various layers of the network. Since the correct class of each segment in the training set is known, an error function was computed as the average difference between the predicted and correct classes for the mini-batch. The gradient of this error function with respect to each of the network’s internal parameters was then used to update the value of the parameter. We used *Adam*, a well-known optimization scheme often used in machine learning, to effect this. Once updated, the network was required to predict the classes of the validation set, the results of which was used to compute the validation error that then was used to monitor the progress of the training. This process was repeated for all 20 mini-batches to complete an *epoch* of training, at the end of which the training dataset was shuffled, split into mini-batches, and the whole sequence repeated. The training continued for a user-chosen number of epochs, which was 7 in our case. If at the end of the seven epochs, both the training and validation errors were below a user-chosen threshold, 1E-3 in our case, the network was accepted as being fully trained.

As for the test data pair, we proceeded in two parallel streams, as illustrated in the flowchart. For Test Data #1, we made a copy of each of the sixteen segments with the full 64-channel data from the one concussed-control pair. This test sample provided a sanity check of the training by ensuring *ConcNet 2* predicted the same score, p(i) as we had gotten in our previous MCCV tests. For Test Data #2, we made 64 replicates of each of the eight data segments for each test participant (i.e., one concussed and one control). In each replicate, we kept the EEG data in one of the channels (taken in turn) unchanged and set the signal in the remaining channels to zero. Each test sample therefore mimicked raw EEG data from a single sensor. This procedure of test sample generation was done for all eight data segments of each participant to yield a total of 2 × 64 × 8 = 1,024 test samples (i.e., 512 concussed and 512 control test samples). It is important to emphasize that these test data were kept separate from the training and validation data for *ConcNet 2*. This was to ensure that the network had no prior knowledge of any of these test data samples.

The relative contribution of each of the 64 channels toward the classification performance of the *ConcNet 2* classifier was ranked using the generated test dataset. For each of the 1,024 data segments in the Test Dataset #2, *ConcNet 2* produced a classification score, as well as the predicted class, n(i,j). Since the true class of each data segment, p(i), in the test set is known, it is possible to assess if using information from a single channel is adequate to correctly predict the class for *both* the concussed and control segments. In order to assess the information content of the channels with respect to classification, each channel was assigned a score of one if *all* sixteen segments (eight from the control and eight from the concussed participant) were correctly classified by *ConcNet 2* using the specific channel as input. Correct classification of all sixteen segments was awarded if and only if the median classification score for the sixteen tests was ≥ 0.9, and the 25th percentile was >0.85. This is a particularly stringent criterion since it only assigns a score of one to channels that accurately predict all sixteen data segments used in the test.

This procedure of training *ConcNet 2* and then testing it on 1,024 test samples was repeated nine times for nine different pairs of control and concussed participants. Since the testing procedure was repeated with nine different control-concussed participant pairs, each channel received a total score between 0 and 9.

We also pursued an additional complementary testing strategy to explore the behavior of *ConcNet 2*, if smaller training and validation sets were used. This scheme involved an identical methodology outlined in the flowchart in Figure 2, except that the test set comprised three pairs of control and concussed participants instead of one pair. The remaining data from 20 concussed and 32 control participants were used to train and validate *ConcNet 2*, and the trained network was then used to classify the data segments of the three control-concussed participant pairs. In this test, each channel received a score of one if *ConcNet 2* correctly classified all 16 segments from one control-concussed participant pair, and this was repeated for the three pairs (the pairing here was random). Hence, each channel received a score between 0 and 3 from the three control-concussed pairs. This entire procedure was repeated three times, for three different sets of three concussed-control pairs of participants, so that the overall score for each channel ranged between 0 and 9.

In order to rank-order the channels based on their impact on the classification performance of *ConcNet 2*, we added the scores from the two tests described above so that each channel’s final score ranged from 0 to 18. We then rank-ordered the channels according to this final score and chose the six top scoring channels for the subsequent tests described under Stage 2. Details of the scoring scheme, and the scores obtained by the top scoring six channels are presented and described in the “Results” section.

It should be noted that for all test data sets and both testing schemes, the *ConcNet 2* network correctly predicted the class labels for both the control and concussed participants when it was presented with the information in all 64 channels. This test served as a benchmark to ensure that the classification performance of *ConcNet 2* was maintained.

In addition to testing the performance of *ConcNet 2* classifier with single-channel inputs, we also analyzed the behavior of the network’s internal parameters (i.e., the weights and biases of the bilinear LSTM units) as well as the output from each of the bilinear LSTM units. The objective of this effort was to identify potential correlations between the values of these parameters and the performance of the network. However, so far no meaningful correlations have been identified.

Stage 2: Investigation of Classification Performance based on a Subset of Top-Ranking Channels.

Having identified the top scoring channels based on their contributions to the classification performance of *ConcNet 2*, we designed a process for evaluating the performance of a network, using raw data from 6 EEG channels for concussion classification. We created a new dataset that consisted of only the six top-scoring channels extracted from the original 64-channel data. This was done for all 8 data segments for all the 23 concussed and 35 control participants.

The architecture of the input layer of *ConcNet 2* was modified to accept six-channel data as input, instead of the full 64 channels. Henceforth, we refer to this modified network as *ConcNet 3*. Given the reduced information available to train the network, it became evident during training and validation that *ConcNet 3* was *overfitting*: During the training phase, the validation error diverged increasingly from the training error, which is a clear sign of overfitting in neural networks. This is a common problem when a sparse dataset is used to train a complex network. In order to control the fitting behavior of the network, we tuned three hyperparameters commonly used to prevent overfitting: We increased dropout fraction to 0.35 from 0.3 for *ConcNet 2*; the initial learning rate was increased from 0.0005 to 0.0008; and the number of training epochs was increased from 7 to 10. Dropout is a commonly used regularization scheme to prevent overfitting by discarding a user-chosen fraction, e.g., 0.35 of the output from the network layer to which it is applied. This has the effect of reducing the complexity of the network. However, as a by-product, the training rate slowed down. To compensate for this, we increased the learning rate. The latter governs the size of the updates applied to the internal parameters by *Adam* during the back propagation in the SGD at the end of each mini-batch. Additionally, we also increased the number of training epochs in order to provide more time for the training to converge to a global minimum in the training error. We refer readers interested in additional details and information about these hyperparameters to Paper I.

The flowchart in Figure 3 illustrates the processing steps comprising our present Stage 2 analysis. Using the 6-channel dataset as input to the re-tuned *ConcNet 3*, we performed Monte Carlo cross validation (MCCV) tests to assess *ConcNet 3*’s performance. As shown on the flowchart, these data were trimmed and segmented into 8 segments, each 90 s long. For the MCCV, we generated an ensemble of 100 clones of *ConcNet 3* networks, each trained validated, and tested with different, randomly selected, training, validation and test datasets, split in the ratio 80:10:10. It should be emphasized that the test dataset was kept completely separate and hidden from the network during training, and used only during testing. Also, the numbers of concussed and control participants in the training dataset were balanced so that the network was not biased against either class. We also decided to retain a validation dataset to ensure that the training proceeded properly with the re-tuned hyperparameters, as explained above.

Each *ConcNet 3* network was trained using the mini-batch SGD method, as described in Stage I of this work. For creating the test dataset, we selected one random segment from each of the 2 concussed and 14 control participants set aside for testing. For each of these 16 test segments, the trained *ConcNet 3* predicted a class, n(i,j). Since the correct class of each segment, q(i) was known, comparing the correct and the predicted classes provided a measure of the accuracy and other statistical performance metrics for *ConcNet 3* for each MCCV cycle. Full details of the MCCV methodology used is described in detail in Paper I. The overall statistical performance measures extracted from the ensemble of MCCV tests are described in “Results” section. These statistical measures form the basis for the performance comparison of *ConcNet 3*, which uses only 6-channel data, with *ConcNet 2* which uses 64-channel data.

## Results

### SCAT Results

In Table 1 we present the demographic information about the participants in this study. All of the concussed participants met the Berlin criteria and exhibited from 4 to 22 SCAT3 symptoms, at the time of testing. The symptoms most frequently reported included irritability, sensitivity to light, dizziness, fatigue, “don’t feel right,” and difficulty concentrating/remembering.

Stage 1: Identification of Channels with Highest Impact on Classification.

**FIGURE 5:**
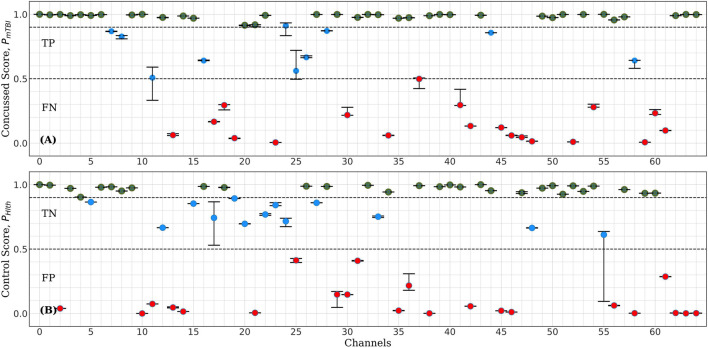
Representative example of *ConcNet 2* test output scores (circle markers), P_Hlth_ and P_Mtbi_, respectively, for one control (bottom) and one concussed (top) participants. The horizontal axis marks the channel number used as input, and the vertical axes correspond to test output scores in the range [0, 1]. The error bars are based on the results of eight data segments per participant, and the circle marker corresponds to the median of these values. Green markers represent channels with medians above 0.9 and 2.5th percentile greater than 0.85. Blue markers correspond to channels with median greater than 0.5 but lower percentiles below 0.85, while red markers correspond to channels with medians less than 0.5 (i.e., misclassifications).

This section presents our results regarding the relative importance of each of the 64 channels of the raw EEG signal in the classification performance of the bilinear LSTM neural network. Figure 5 shows typical classification scores for one of the nine control-concussed participant pairs, as described in the “Materials and Methods” section (Stage 1). In each of these tests the trained *ConcNet 2* network classified all eight data segments of a pair of concussed and control participants. For each test sample (corresponding to one of the eight segments from each participant), the network outputs a concussed score, P_mTBI_ in the range 0–1 and the complementary control score, P_Hlth_ = 1-P_mTBI_. For classification purposes, we use a discrimination threshold equal to 0.5. Hence, if the predicted score, P_mTBI_ ≥ 0.5, the sample is classified as concussed. Similarly, a sample is classified as control if P_Hlth_ is greater than 0.5. The top panel of Figure 5 shows the output scores (P_mTBI_ vertical axis) of *ConcNet 2* obtained by using each of the 64 channels (marked along the horizontal axis) for the samples drawn from the concussed participant. Channel 0 represents the benchmark case which includes all 64 channels in order to verify the expected correct classification of *ConcNet 2* in this case. Since each concussed participant contributes eight data segments, we obtained the median and the 2.5th and 97.5th percentiles of the output scores. Channels for which the median value exceeded 0.9, and the 2.5th percentile 0.85, are marked with a green circle in the plot. By selecting channels with high median values and low scatter (as indicated by a relatively high value of the lower percentile), we identify channels with high information content with respect to classification. On the other hand, if only the median value exceeds 0.5 but the 2.5th percentile does not exceed 0.85, the channel is shown with a blue marker to indicate that this channel provided the correct prediction at least half of the times. If, however, the median fell below the threshold of 0.5, the channel is shown with a red marker to flag an incorrect prediction at least half of the time. The bottom panel presents the same information for data segments drawn from the control participant. Hence, in this case the output scores on the vertical axis correspond to P_Hlth_.

For the test results represented in Figure 5, 35 of the 64 channels fell in the high scoring category (green points), which marks their ability to correctly predict the concussed sample with a high degree of confidence. Similarly, 29 channels fell in the high scoring category for the control case. When the concussed and control scores were combined, 20 channels emerged in the high scoring category. These 20 channels were assigned a score of one for this test, and the remaining channels were given a score of 0. Figure 4 shows the combined classification test scores for the control and the concussed pair, whose individual scores are shown in Figure 5. Notice that the median and the error bars in this plot are based on sixteen segments (eight from the control and eight from the concussed individual); this is the reason that the error bars are wider than those shown in Figure 5.

The assignment of scores to channels was repeated for all eighteen test data sets described in “Materials and Methods” section. Channels which scored 10 or higher were selected as the top scorers for further investigation in Stage 2. The six top-scoring channels were those numbered 1, 8, 32, 39, 43, and 54, with overall scores, 10, 10, 16, 14, 16, and 10, respectively. Data from these six channels were extracted to form a modified dataset, which was used for Monte Carlo cross validation studies in Stage 2. The schematic in Figure 1 shows the locations of the six top-scoring channels (marked by yellow squares).

Stage 2: Investigation of Classification Performance based on a Subset of Top-Ranking Channels using *ConcNet 3*.

**FIGURE 6:**
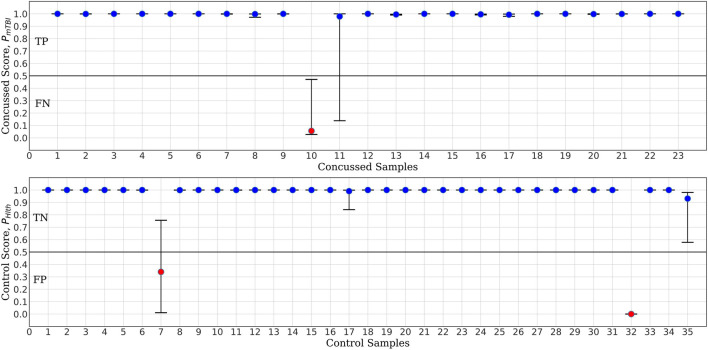
*ConcNet 3* MCCV classification test output scores (circle markers), i.e., P_Hlth_ and P_mTBI_, respectively, for control (bottom) and concussed (top) participants. The horizontal axis marks the different participants in each group, and the vertical axes correspond to test output scores in the range [0, 1]. The error bars are based on the classification results of 100 Monte Carlo cross validation experiments. Blue markers correspond to participants who were correctly classified at least 50% of the time, while red markers correspond to misclassified participants (median classification score less than 0.5).

The MCCV test results for *ConcNet 3* based on an ensemble of 100 cross validation experiments using as input the six top scoring channels are shown in Figure 6. Each data point in the upper panel represents one of the 23 concussed participants in our dataset, and the data points in the lower panel correspond to the available 35 control participants. The random training—validation—test split used in MCCV means that in each cross validation experiment, any concussed and control participants may be included in the test set. The concussed or the control score assigned by the network each time a data segment from the specific participant is tested is saved. Since each sample is included in the test set several times during the 100 cross validation experiments, the medians as well as the 25th and 75th quartiles of the scores can be calculated from the Monte Carlo ensemble. The blue points in the upper panel of Figure 5 correspond to concussed participants who were correctly classified (True Positives, TP) at least 50% of the time. The high median values and small interquartile range of these participants indicate a high degree of confidence in their classifications by the ensemble of networks. There is only one participant, shown by the red circle, which was consistently misclassified (False Negative, FN), while the score of one participant exhibits a large scatter. The lower panel shows the ensemble-based classification scores for the 35 control participants. Two of them were consistently misclassified (False Positives, FP), leaving 33 participants in the True Negative (TN) category.

**FIGURE 7:**
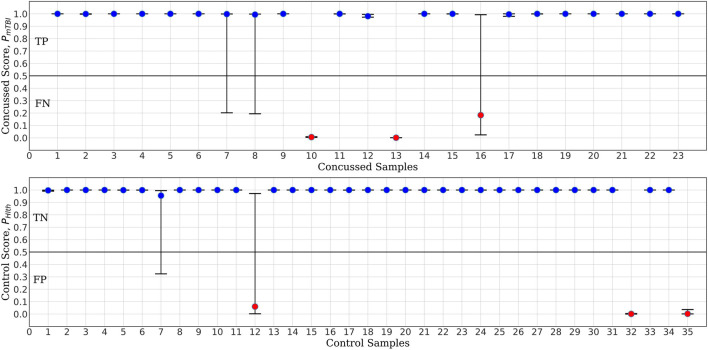
*ConcNet 2* MCCV classification test output scores (circle markers), i.e., P_Hlth_ and P_mTBI_, respectively, for control (bottom) and concussed (top) participants. These results are based on information from all 64 EEG channels. The horizontal axis marks the different participants in each group, and the vertical axes correspond to test output scores in the range [0, 1]. The error bars are based on the classification results of 100 Monte Carlo cross validation experiments. Blue markers correspond to participants who were correctly classified at least 50% of the time, while red markers correspond to misclassified participants (median classification score less than 0.5).

For the purpose of benchmark comparison, Figure 7 shows the performance of *ConcNet 2* in a similar MCCV test, with the network using the same architecture and hyperparameters as described in Paper I. The input data for *ConcNet 2* are the original 64-channel, raw EEG data. In this case, the upper panel (representing the concussed participants), shows three FN and two participants with significant scatter, while the remaining 18 samples fall in the TP category. In the case of control participants, shown in the lower panel, there are three FP and two samples with considerable scatter, while the remaining 30 samples are all TN with small scatter.

Based on the True Positive and Negative rates, as well as the False Positive and Negative statistics obtained from the MCCV tests, we computed the standard performance metrics for both networks (see Paper I for the definitions of these metrics). The median and the upper and lower quartiles of these performance metrics are given in Tables 2, 3 for *ConcNet 3* and *ConcNet 2*, respectively. The accuracy of *ConcNet 3* is 94.4% (with upper and lower quartiles of 88.9 and 97.2%) which are comparable to those of *ConcNet 2*. The recall of both networks is high, justifying that both networks perform equally well in identifying concussed cases. The presence of the misclassified control participants is indicated by the lower precision for both networks. The false positive and negative rates are low for both networks.

**FIGURE 8:**
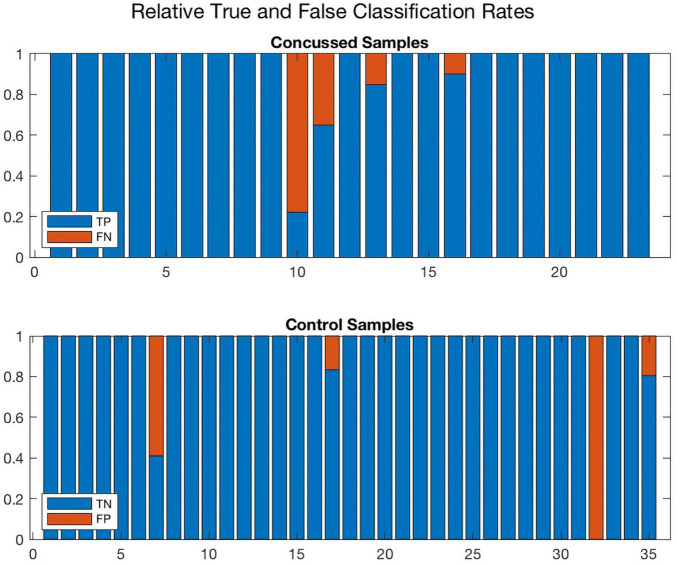
A bar plot representation of the Monte Carlo cross validation test results obtained by means of the *ConcNet 3* ensemble of networks (using the six top-scoring channels as input) for the concussed (top) and control (bottom) participants. The blue and red regions in each column illustrate the relative fractions of times that a participant was properly or wrongly classified by the MCCV ensemble. This figure is complementary to Figure 5 which shows the *ConcNet 3* median and quartile scores per participant. Two control participants and one concussed participant tended to be systematically misclassified by the networks in the ensemble.

**FIGURE 9:**
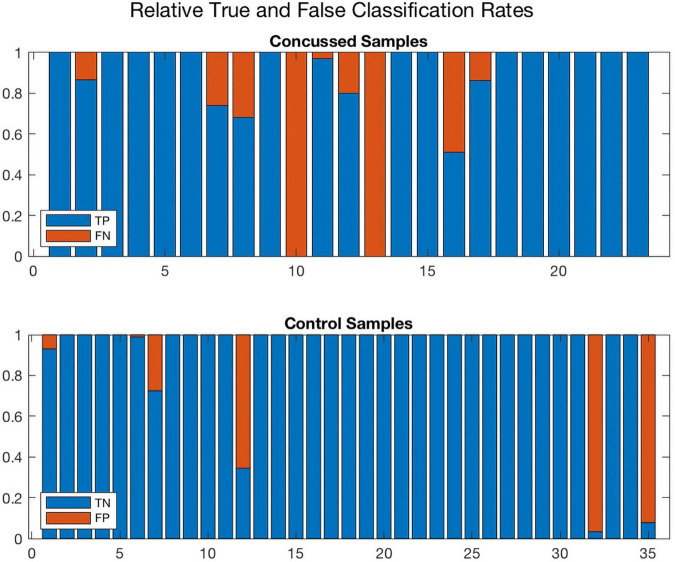
A bar plot representation of the Monte Carlo cross validation test results obtained by means of the *ConcNet 2* ensemble of networks (using all 64 channels as input) for the concussed (top) and control (bottom) participants. The blue and red regions in each column illustrate the relative fractions of times that a participant was properly or wrongly classified by the MCCV ensemble. This figure is complementary to Figure 4 which shows the *ConcNet 2* median and quartile scores per participant. Three control and two concussed participants were systematically misclassified by the networks in the ensemble.

Further, Figures 8, 9 compare the performance of *ConcNet 3* and *ConcNet 2* by means of bar plots, which summarize the results of the MCCV tests for the control and concussed participants. Again, the performance of the two classifiers is quite similar, with *ConcNet 3* even achieving lower misclassification rates than *ConcNet 2*.

**FIGURE 10:**
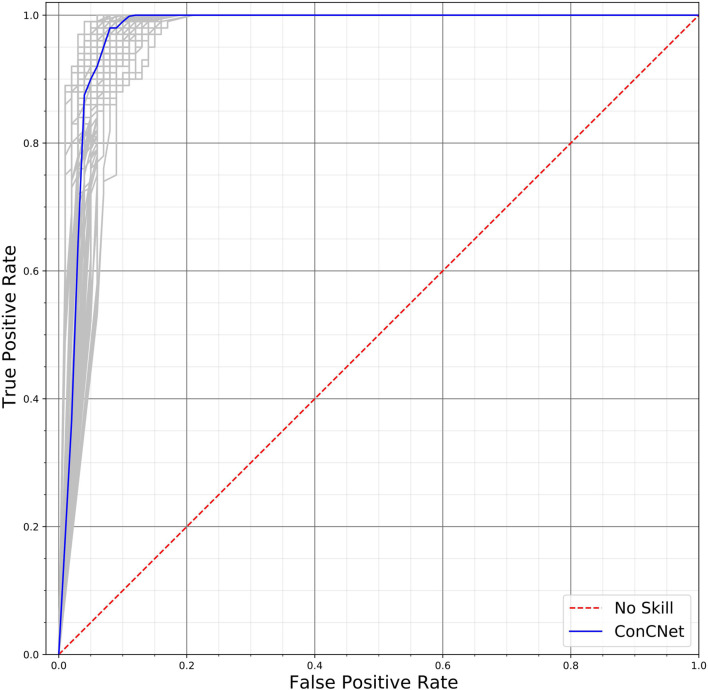
Receiver operating characteristic (ROC) curves for the 100 *ConcNet 3* networks used in MCCV. The gray curves show the results for each of the 100 networks and the blue curve shows the median. The ensemble median Area Under the Curve (AUC) is 0.971 (the 25% and the 75% percentile are 0.964 and 0.978, respectively).

**FIGURE 11:**
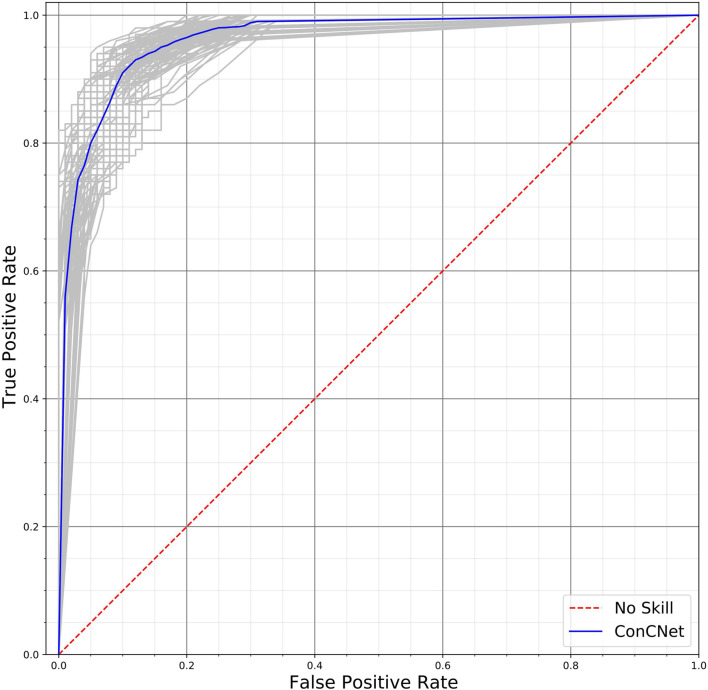
Receiver operating characteristic (ROC) curves for the 100 *ConcNet 2* networks used in MCCV. The gray curves show the results for each of the 100 networks and the blue curve shows the median. The ensemble median Area Under the Curve (AUC) is 0.961 (the 25% and the 75% percentile are 0.952 and 0.969, respectively).

A different way of assessing the performance of the two networks is by means of the Receiver Operating Characteristic (ROC) curves, shown in Figure 10 (*ConcNet 3*) and Figure 11 (*ConcNet 2*). In ROC curves the true positive rate (TPR) of a binary classifier is plotted against the False Positive Rate (FPR) for different values of the threshold that discriminates between concussed and control samples. Thus, ROC curves portray the classification ability of binary classifiers for different thresholds. A perfect classifier corresponds to the point with TPR = 1 and FPR = 0 (in the upper left corner of the ROC plot), while a random classifier would lie along the diagonal line of no-discrimination defined by the equation TPR = FPR. The comparison of the ROC curves in Figures 10, 11 show that both networks perform well.

*ConcNet 2* and *ConcNet 3* perform significantly better than a random classifier, as evidenced by the fact that all the ROC curves (obtained from the MCCV ensemble) concentrate above the no-discrimination line and cluster around the upper left corner in ROC space. In fact, the ROC curves obtained by *ConcNet 3* rise more sharply toward the top of the ROC space, indicating better classification performance. A scalar measure that summarizes the information of the ROC curve is the Area Under the Curve (AUC). A classifier with no better accuracy than chance would be expected to have an AUC of 0.5, while an AUC of 1 corresponds to a classifier with perfect accuracy. The median AUC score of *ConcNet 3* was 0.971 (with lower and upper quartiles 0.964 and 0.978), while the corresponding values for *ConcNet 2* were 0.961, quartiles 0.952 and 0.969.

## Discussion

We have recently shown that a deep learning LSTM-based recurrent neural network was able to distinguish between non-concussed and acute post-concussed adolescent athletes using only 90 s long samples of raw, resting state, EEG data using 64 channel data with an accuracy of >90% (Thanjavur et al., 2021). Here, we present novel results showing that a minimal subset of 6 channels (from the original 64 channel dataset) can achieve similar results for concussion classification to identify post-concussed individuals within 1 month of injury. This is the first proof of concept showing that a deep learning network can be trained to recognize individuals with concussion using only 6 channels associated with the resting state condition.

The key question that we address in this paper is whether the information from a reduced set of channels is sufficient for the neural network to correctly classify concussion. The most thorough way of performing this analysis would be to consider as input to the neural network subsets of M channels, where M is an integer between 1 and 64, and to rank the performance of the network for each input. We would then need to investigate all the possible combinations involving M channels, i.e., C (N, M) = N!/M! (N-M)!, for all M = 1, 2, …, 64. This implies a very large number of possible configurations for values of M that are not close to either 1 or 64. In order to circumvent this combinatorics dimensionality curse we opted to examine the impact of each channel independently of the others. Specifically, we started with our previous 64-channel classifier (*ConcNet 2*), and we explored the classification performance based on a single channel at a time, calculating an accuracy score for each of the 64 channels taken one-by-one. We then rank-ordered the channels and retained all channels with accuracy above a selected threshold. This process identified six top-scoring channels. As stated in “Materials and Methods” section, this approach does not account for potential interactions between channels. However, for our purposes an “optimal solution” is not necessary. Our objective was simply to determine a reduced subset of channels that would give comparable classification performance as *ConcNet 2*. For a perspective on the computational gains afforded by the one-channel-at-a-time strategy, consider that testing all possible combinations of six out of 64 channels would require testing more than 74 million configurations.

A key finding of this study is that the top channels picked one at a time, were subsequently used successfully by the network to distinguish between the two groups and turned out to have functional significance. The 6 channels (i.e., channel #s: 1, 8, 32, 39, 43, and 54) correspond to three sensors in the right frontal regions: (R) anterior-frontal, (R) mid-frontal, (R) central-frontal, and three in the posterior regions: (L) occipital, (R) occipital, and (R) parieto-occipital. An intriguing question is why these particular sensors emerged as the top ranked and what is their significance in relation to known pathophysiology of concussion. While it is well established that the location of EEG sensors does not have a 1:1 correspondence with underlying neuroanatomy (Schoffelen and Gross, 2009), we consider the relevance of these channels broadly in relation to findings from the concussion literature.

Diffusion tensor imaging (DTI) shows that the stretching and tearing of the brain tissue, caused by the acceleration and deceleration forces acting upon the head during concussive impact, result in a diffuse disconnection pattern that affects the white matter architecture of the brain. Several white matter tracks are typically reported in studies involving child and youth concussions including the corona radiata, the genu of the corpus callosum, the fornix and the cingulum, the corticospinal tract, the internal capsule, and the superior longitudinal fasciculus (Borich et al., 2013; Yallampalli et al., 2013; Virji-Babul et al., 2014; Yuan et al., 2015; Manning et al., 2017; Murdaugh et al., 2018).

Damage to such white matter pathways and traumatic axonal injuries disrupts information flow across brain areas (Caeyenberghs et al., 2017) and frontal regions of the brain are particularly affected in children and youth. Using resting state EEG, we showed significant increases in the functional connectivity in areas corresponding to the right inferior frontal gyrus and the right dorsolateral prefrontal cortex (Virji-Babul et al., 2014) in adolescents with concussion. Using fMRI we noted increased functional connectivity primarily concentrated in the right frontal region within the executive function network (Borich et al., 2015). Similarly, Newsome et al. (2016) found that asymptomatic adolescent athletes demonstrated increased connectivity (relative to a cohort of high school athletes with orthopedic injuries) between the posterior cingulate cortex and the ventral lateral prefrontal cortex.

More recently, we studied changes in effective connectivity (information flow between brain regions) and found that adolescents with subacute concussion show distinct changes in the pattern of information flow within the frontal regions during resting state (Hristopulos et al., 2019). We observed strong information flow between the fronto-polar midline (FpM) and the mid-frontal region in the concussed group that were not present in the controls. In addition, there were strong bidirectional connections in information flow between the FpM and right frontal regions (FR) while the controls showed lateralized information flow in the left frontal regions of the brain. These changes suggest a “re-routing” of information flow as a function of brain injury and highlight that increased connectivity in the (R) frontal region is a significant neuroplastic response to injury. Numerous studies have reported a frontal and specifically, prefrontal vulnerability to brain injury. It is possible that such changes in the frontal regions of the concussed brain may have been detected by the neural network.

While the frontal regions have been specifically associated with adolescent concussion, there are a number of other regions that show both increased and decreased functional connectivity and coupling in concussion. For example, Safar et al. (2021) recently found changes in frontal, temporal and occipital brain regions in children and adolescents who are in the chronic stages of mild traumatic brain injury, suggesting that in addition to the frontal regions, there are at least two other brain regions of interest.

While it is tempting to make an association between known regions of the brain that are involved in concussion and selecting specific EEG sensors, it is premature at this stage to argue that there is a unique subset of EEG channels or brain regions that are ideal for classifying concussion. We do not know what features the deep learning network is focusing on. Additional testing with a more diverse group of participants will be required to identify a unique subset of channels that might be useful for concussion diagnosis.

In addition, two outstanding questions still remain. First, is it possible to determine the degree of severity following the initial brain injury using the neural network? Second, how does the neural network respond to changes in EEG related to recovery? Identifying the severity of concussion and determining clinical recovery are two of the most challenging issues for clinicians as well as researchers in this field. In principle, one can contemplate expanding the approach described in the present paper and leverage unsupervised learning to cluster subjects with similar degrees of brain changes but a clear understanding of what these changes mean for brain health remains elusive. This is in part due to the fact that diagnosis continues to be based primarily on the patient’s subjective reports of signs and symptoms and importantly, symptoms do not directly correlate to the underlying changes in brain structure and function (Manning et al., 2017; Churchill et al., 2019). In addition, neuroimaging studies find that the brain’s response to a mild brain injury varies considerably from participant to participant both in the acute phase as well as in the brain’s recovery trajectory (Churchill et al., 2017, 2020). The resolution of both these questions will require a large sample of data that encompasses the full range of clinical diversity in signs and symptoms as well as the full range of brain changes present at the time of injury and over the course of recovery. Collecting data on a longitudinal, diverse sample of individuals will provide much needed information to achieve our goal of designing a portable, easy to use EEG system with a subset of a small number of sensors to identify both severity and the range of recovery trajectories.

### Limitations

The study described here has two main limitations. The first of these is that the EEG data set used to train and test the network is relatively small and the data was acquired only from male adolescent athletes. As the COVID-19 restrictions are gradually being lifted, we will actively begin recruiting a more diverse sample of participants to evaluate the effects of sex, age and stage of recovery.

The second limitation has been discussed above and is related to the method used to identify the subset of the most important channels for classification. In particular, the identification of each channel’s impact on classification is performed independently of the other channels. However, we believe that this methodological choice does not significantly affect the “optimal” subset of channels. This assessment is supported by the fact that the top-ranking channels thus determined admit a neurophysiological interpretation which agrees with our current understanding of concussion.

## Summary

In summary, our study demonstrates for the first time that a minimal subset of 6 channels (from the original 64 channel dataset) can achieve similar results for concussion classification to identify post-concussed individuals within 1 month of injury. This is the first proof of concept showing that a deep learning network can be trained to recognize individuals with concussion using only 6 channels associated with the resting state condition. While these channels may not be the optimal subset of channels, they do correspond with current understanding of the neurophysiological changes in concussion. Further work is needed to evaluate the algorithm with a diverse group of individuals.

## Data Availability Statement

The original contributions presented in the study are included in the article/supplementary material, further inquiries can be directed to the corresponding author/s.

## Ethics Statement

The studies involving human participants were reviewed and approved by the University of British Columbia Clinical Research Ethics Board (Approval Number: H17-02973). Written informed consent to participate in this study was provided by the participants legal guardian/next of kin.

## Author Contributions

AB, DH, and NV-B conceived the investigation. NV-B prepared the data. AB, KT, and DH contributed to the design of the network. KT trained and tested the network. KT and KMY provided support on network development. AB, NV-B, KT, DH, and KMY interpreted the results and substantially revised the manuscript. NV-B, KT, and DH drafted the manuscript. All authors reviewed the manuscript.

## Conflict of Interest

The authors declare that the research was conducted in the absence of any commercial or financial relationships that could be construed as a potential conflict of interest.

## Publisher’s Note

All claims expressed in this article are solely those of the authors and do not necessarily represent those of their affiliated organizations, or those of the publisher, the editors and the reviewers. Any product that may be evaluated in this article, or claim that may be made by its manufacturer, is not guaranteed or endorsed by the publisher.
